# Anti-Leukemic Effects on a U937 Cell Line of Fresh and Steamed Chinese Kale Juice and Their Pro-Apoptotic Effects via a Caspase-Dependent Pathway

**DOI:** 10.3390/foods12071471

**Published:** 2023-03-30

**Authors:** Siriphorn Pungpuag, Somchai Boonpangrak, Yaneenart Suwanwong

**Affiliations:** 1 Clinical Hematology Sciences Program, Faculty of Allied Health Sciences, Chulalongkorn University, Bangkok 10330, Thailand; siriphorn.kn@gmail.com; 2 Center for Research and Innovation, Faculty of Medical Technology, Mahidol University, Nakhon Pathom 73170, Thailand; somchai.boo@mahidol.ac.th; 3 Department of Clinical Microscopy, Faculty of Allied Health Sciences, Chulalongkorn University, Bangkok 10330, Thailand

**Keywords:** apoptosis, *Brassica*, cancer, glucosinolate, isothiocyanate, kale, leukemia

## Abstract

Chinese kale is a vegetable belonging to the family Brassicaceae in which members of this family produce unique metabolites called glucosinolates and isothiocyanates. These substances have been found to exhibit many benefits to human health. This study aimed to investigate and compare the contents of glucosinolates and isothiocyanates, and the anti-leukemic activity of fresh and steamed Chinese kale juice (CKJ). Cell viability and proliferation activity of U937 cells treated with CKJ were determined. Cell apoptosis and alterations of apoptosis-related protein expression were studied. Results showed that CKJ significantly decreased the viability of leukemic cells and inhibited cell proliferation in a dose- and time-dependent manner. After treatment with 5% *v*/*v* fresh and steamed CKJ for 24 h, the percentage of apoptotic cells increased to 53% and 36%, respectively. Increased amounts of cleaved caspase-3 in U937 cells treated with CKJ were observed, indicating that CKJ can trigger apoptotic cell death through a caspase-dependent pathway. Fresh CKJ was found to be more effective than steamed CKJ in suppressing cell survival and inducing cell apoptosis. The results suggest that Chinese kale possesses an anti-leukemic potential and could be further developed for cancer therapy and prevention. However, thermal cooking could reduce its beneficial function.

## 1. Introduction

Chinese kale (*Brassica oleracea* var. *alboglabra*) is a nutrient-rich vegetable belonging to the family Brassicaceae, formerly known as the family Cruciferae. Members of this family include several types of edible plants known as cruciferous vegetables. They have been recognized as sources of various phytochemicals and nutraceuticals, e.g., alkaloids, tannins, phenolic compounds, flavonoids, carotenoids, and vitamins [1]. There is strong evidence that consuming cruciferous vegetables can reduce cancer risk and improve cancer patients’ survival [2,3,4,5]. Notably, cruciferous vegetables produce unique secondary metabolites called glucosinolates and isothiocyanates, which have proven to be the dominant contributors to the anticancer activity of *Brassica* vegetables [6,7,8]. Glucosinolates are a group of nitrogen- and sulfur-containing compounds biosynthesized from amino acids. Isothiocyanates constitute the major products of myrosinase-catalyzed glucosinolate hydrolysis and are responsible for the pungent aromas and spicy or bitter tastes of cruciferous vegetables [9]. Under normal conditions, plants store myrosinase enzymes and glucosinolates through compartmentalization. For example, when plant tissues are damaged by chopping or chewing, the glucosinolates are exposed to myrosinase and converted to isothiocyanates. However, extreme heat (>70 °C) can destroy the myrosinase enzyme, leaving the glucosinolates unaltered [10]. The beneficial effects of glucosinolates and isothiocyanates on human health have been reported. These include anti-oxidative, anti-inflammatory, antimicrobial, anticancer, and neuroprotective activities [6,11,12]. Most studies have concentrated on vegetables commonly consumed in western countries, e.g., broccoli, Brussels sprouts, and turnips. However, less attention has been paid to vegetables used in Asian cuisine, namely Chinese kale. A previous study showed that Chinese kale possesses anti-oxidative activity [13]. However, its anticancer properties have not been explored to date.

Leukemias are a group of life-threatening malignant disorders of the blood and bone marrow. Currently, most patients with leukemia are treated with chemotherapy, and some patients may receive bone marrow transplants. Although these treatments are efficient, they often result in adverse effects. The development of resistance to chemotherapeutic drugs also negatively affects therapeutic outcomes [14,15]. Therefore, many researchers keep searching for leukemia treatments that are based on cytotoxic substances that do not produce side effects. For more than two decades, plant-derived compounds have been studied and proven to be effective against cancers with minimal toxicity [16].

This study aimed to investigate whether Chinese kale has anti-leukemic activity and to compare the levels of glucosinolates and isothiocyanates and the anti-leukemia potential of fresh and steamed Chinese kale juice (CKJ). The mechanism involving the anti-leukemic properties of Chinese kale was also elucidated.

## 2. Materials and Methods

### 2.1. Preparation of Chinese Kale Juice

The organic Chinese kale (*Brassica oleracea* var. *alboglabra*) was bought from a supermarket in Bangkok, Thailand, in January 2019. Chinese kale leaves from several stalks and bundles were gathered together until they weighed 500 g. Leaves without petioles were thoroughly rinsed with tap water, air-dried and randomly divided into 2 equal groups. One group was chopped to induce myrosinase hydrolysis and passed through a mechanical juicer. The juice obtained was labeled fresh CKJ. Another group was not chopped but brought to steam over boiling water for 10 min before passing through a mechanical juicer. The juice obtained was labeled steamed CKJ. Fresh and steamed CKJ were stored at −20 °C until analysis. CKJ was filtered through a 0.2 μm syringe filter (Minisart^®^; Sartorius, Goettingen, Germany) before use.

### 2.2. Determination of Total Glucosinolate Contents

The total glucosinolate contents of the juice were determined using the ferricyanide-based spectrophotometric method [17], with some modifications. Briefly, CKJ was incubated at 80 °C for 20 min to inhibit myrosinase activity and then centrifuged at 12,000 rpm for 10 min at 4 °C. Four hundred fifty microliters of the supernatant were used for alkaline hydrolysis with an equal volume of 2 M NaOH for 30 min and then neutralized with 70 μL of concentrated HCl. The neutralized sample was incubated with an equal volume of 2 mM potassium ferricyanide in 0.4 M phosphate buffer pH 7.0 for 2 min. The absorbance was measured at 420 nm using a spectrophotometer. The total glucosinolate content of the extract was quantified using the standard curve of sinigrin (Figure S1).

### 2.3. Determination of Total Isothiocyanate Contents

The total isothiocyanates of the juice were determined using the previously reported method. Firstly, the 1,3-benzodithiole-2-thione standard was prepared as described by Kristensen et al. [18]. Briefly, 5.6 mmol of 1,2-benzenedithiol was dissolved in 12 mL methanol and 24 mL potassium phosphate buffer (pH 8.5). An equal volume of propyl isothiocyanate (9.9 mmol) in methanol was added dropwise to the previous solution under constant stirring. The mixture was incubated overnight at room temperature. The mixture was extracted 3 times with chloroform. Then, chloroform was removed using a rotary evaporator. Purification of 1,3-benzodithiole-2-thione was carried out by thin-layer chromatography. Sample analyses were performed using the method of Fusari et al. [19]. Briefly, CKJ was incubated at 37 °C for 2 h to complete the glucosinolate hydrolysis and then centrifuged at 10,000 rpm for 2 min. Two hundred fifty microliters of the hydrolyzed sample were mixed with 250 μL potassium phosphate buffer (pH 8.5) and 500 μL of 1,2 benzenedithiol (10 mM) in propanol. The mixture was incubated at 65 °C for 2 h and centrifuged at 14,000 rpm for 5 min. The supernatant was filtered and then analyzed using HPLC (Figure S2).

### 2.4. Cell Culture

U937 cells were obtained from the American Type Culture Collection (ATCC CRL-1593.2; Rockville, MD, USA) and maintained in RPMI 1640 medium containing 10% heat-inactivated fetal bovine serum (Gibco; Thermo Fisher Scientific, Inc., Waltham, MA, USA) at 37 °C in a humidified 5% CO_2_ incubator.

### 2.5. Cell Viability Analysis

U937 cells (3 × 10^4^ cells/well) were seeded into 96-well plates and treated in triplicate with either fresh or steamed CKJ (0.625, 1.25, 2.5, and 5% *v*/*v*) for 24, 48, and 72 h. At the indicated time intervals, cell viability was determined using a trypan blue dye exclusion assay using a hemocytometer under the light microscope. For controls, normal saline (0.9% NaCl) was added instead of CKJ. Three independent experiments were performed.

### 2.6. Cell growth Inhibition Analysis

The XTT assay (Sigma–Aldrich, Merck KGaA, Darmstadt, Germany) was conducted to investigate cell growth. U937 cells (3 × 10^4^ cells/well) were seeded into 96-well plates and treated in triplicate with either fresh or steamed CKJ (0.625, 1.25, 2.5, and 5% *v*/*v*) for 24, 48, and 72 h. Then, the XTT assay was performed according to the manufacturer’s recommended protocol. Briefly, the XTT solution was mixed with PMS and added to each well, followed by incubation at 37 °C in a humidified 5% CO_2_ incubator for 3 h. The absorbance was determined at 450 nm using a microplate reader (Synergy MX; BioTek Instruments, Inc., Winooski, VT, USA). Three independent experiments were performed.

### 2.7. Cell Cycle Analysis

The cell cycle distribution was investigated using flow cytometry analysis. U937 cells treated with different concentrations of fresh or steamed CKJ for 24, 48, and 72 h were washed with cold PBS and fixed in cold 70% ethanol for 18 h at 4 °C. After fixation, cells were washed with cold PBS, and cell pellets were stained with 50 µg/mL propidium iodide (PI; Bio Basic Canada Inc., Markham, CA, USA) in the presence of 100 µg/mL RNaseA (Sigma, St. Louis, MO, USA) for 1 h at 4 °C. PI fluorescence intensity was determined using flow cytometry (FACS Calibur; BD Biosciences, San Jose, CA, USA). For controls, normal saline (0.9% NaCl) was added instead of CKJ. The percentages of the population in G0/G1, S, and G2/M phases of the cell cycle were determined using CellQuest software (BD Biosciences, San Jose, CA, USA). Three independent experiments were performed.

### 2.8. Cell Apoptosis Analysis

The apoptosis kit (ImmunoTools, Friesoythe, DE) was used to analyze cell apoptosis in U937 cells (2 × 10^5^ cells) treated with fresh and steamed CKJ (1.25, 5 and 20% *v*/*v*). For controls, normal saline (0.9% NaCl) was added instead of CKJ. Cells were harvested at different incubation times and resuspended in 90 µL binding buffer. Fluorescein isothiocyanate (FITC)-conjugated annexin V and PI reagent staining was performed at the concentrations and times recommended by the manufacturer. Stained cells were analyzed using a flow cytometer (FACS Calibur; BD Biosciences, San Jose, CA, USA), and data were analyzed using CellQuest software (BD Biosciences).

### 2.9. Western Blot Analysis

U937 cells were seeded in a cell culture dish at 2 × 10^5^ cells/mL and treated with 5% *v*/*v* fresh or steamed CKJ for 72 h. For controls, normal saline (0.9% NaCl) was added instead of CKJ. Cell lysis was performed using the lysis buffer (Boster Biological Technology, Pleasanton, CA, USA) plus protease inhibitor cocktail (Cell Signaling Technology, Danvers, MA, USA) for 30 min on ice. The lysates were clarified using centrifugation at 13,000× *g* for 10 min at 4 °C. Protein concentration was determined using a BCA protein assay kit (Bio-Rad, Hercules, CA, USA). The lysates were subjected to sodium dodecyl sulfate-polyacrylamide gel electrophoresis (SDS-PAGE) on a 4% stacking and a 12% separating gel for protein separation and then blotted onto a nitrocellulose membrane. The membrane was soaked in 5% bovine serum albumin (BSA; Bio Basic Canada Inc., Markham, CA, USA) in Tris-buffered saline with Tween 20 (TBST; 20 mM Tris-base, 150 mM NaCl, 0.1% Tween20, pH 7.4) for 1 h at room temperature, and then incubated with primary antibodies against B-cell lymphoma 2 protein (Bcl-2) (1:1000; Cell Signaling Technology), Bcl-2-associated X protein (Bax) (1:1000; Cell Signaling Technology), cleaved caspase 3 (1:1000; Cell Signaling Technology), and β-actin (1:2000; Cell Signaling Technology) at 4 °C overnight. Subsequently, the membrane was washed three times with Tris-buffered saline with 0.1% Tween^®^ 20 Detergent (TBST) and incubated with secondary antibodies conjugated with horseradish peroxidase (1:1000; Cell Signaling Technology) at room temperature for 1 h. Finally, the membrane was washed three times with TBST, and bands were visualized using an enhanced chemiluminescence (ECL) detection reagent kit (Bio-Rad, Hercules, CA, USA). Table S1 demonstrates antibody lists used in this study.

### 2.10. Statistical Analysis

All experiments were performed in triplicate. The data were expressed as the mean ± standard error. Statistical analysis was performed using GraphPad Prism version 8.0.2 (GraphPad Software Inc., San Diego, CA, USA). An independent t-test was used to evaluate the differences in the mean of total glucosinolates and total isothiocyanates between fresh and steamed CKJ. Effects of CKJ on U937 cells were compared using the analysis of variance (ANOVA) followed by Tukey’s multiple comparison tests. *p*-values less than 0.05 were considered statistically significant.

## 3. Results

### 3.1. Total Glucosinolate and Total Isothiocyanate Contents of the CKJ

The total glucosinolate and total isothiocyanate contents of the juice extracted from fresh and steamed Chinese kale leaves were determined. The results are shown in Table 1. The steamed Chinese kale was prepared by steaming for 10 min to inhibit myrosinase activity. The results showed that there is no difference in total glucosinolate contents between fresh and steamed CKJ and total isothiocyanate contents in steamed CKJ are slightly higher than in fresh CKJ.

### 3.2. Effects of CKJ on the Viability and Growth of U937 Cells

**Figure 1:**
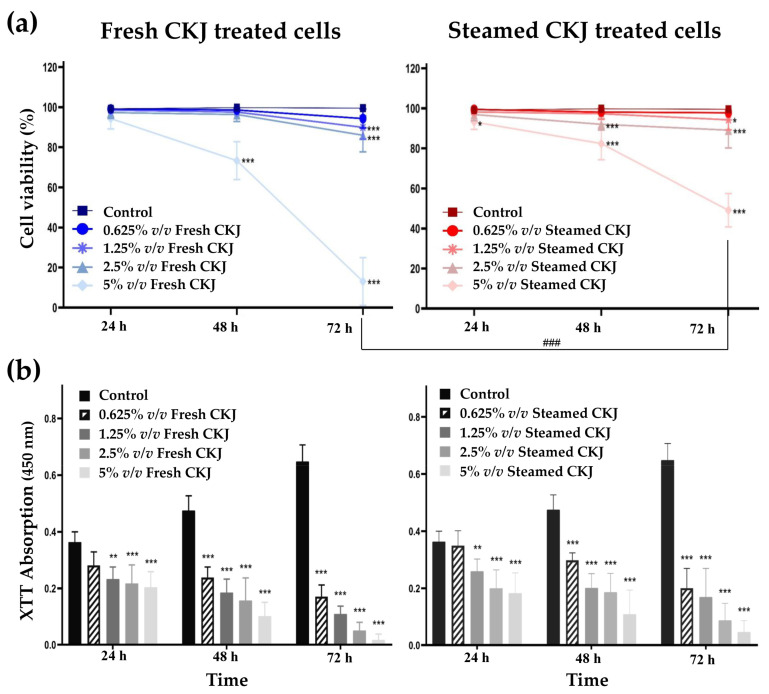
Effects of CKJ on U937 cell survival (**a**) and cell growth (**b**). Cells were exposed to different concentrations of fresh and steamed CKJ for 24, 48, and 72 h. The significance level of the tested sets was compared with a control (normal saline) set (* *p* = 0.05, ** *p* = 0.01 and *** *p* < 0.001) and fresh CKJ-treated cells were compared with steamed CKJ-treated cells (### *p* < 0.001). Data were representative of three independent experiments.

The myelomonocytic leukemic cell line U937 cells were treated with different concentrations of fresh and steamed CKJ for different time intervals up to 72 h, and the cell viability and growth were determined. The results showed that fresh and steamed CKJ decreased the viability of U937 cells in a concentration- and time-dependent manner (Figure 1a). At 48 and 72 h, fresh CKJ showed a higher effect than steamed CKJ on cell viability, especially at 72 h, 5% *v*/*v* of fresh CKJ decreased cell viability to 13 ± 11.9%, whereas treatment with 5% *v*/*v* steamed CKJ decreased cell viability to 49 ± 8.3%. Neither fresh nor steamed CKJ at 0.625% *v*/*v* affected cell viability, even after treatment for 72 h. The results of the cell growth inhibition analysis showed that both fresh and steamed CKJ could inhibit cell growth in a concentration- and time-dependent manner (Figure 1b). The growth inhibitory effect was clearly observed for every incubation time, although the effect of the 0.625% *v*/*v* concentration was not significant at 24 h, and when compared between fresh and steamed CKJ, the effect was not significantly different. These results indicated that the juice from Chinese kale exerted anti-leukemic effects on U937 cells by disrupting cell proliferation and at high concentrations causing cell death.

### 3.3. Effects of CKJ on the Cell Cycle

**Figure 2:**
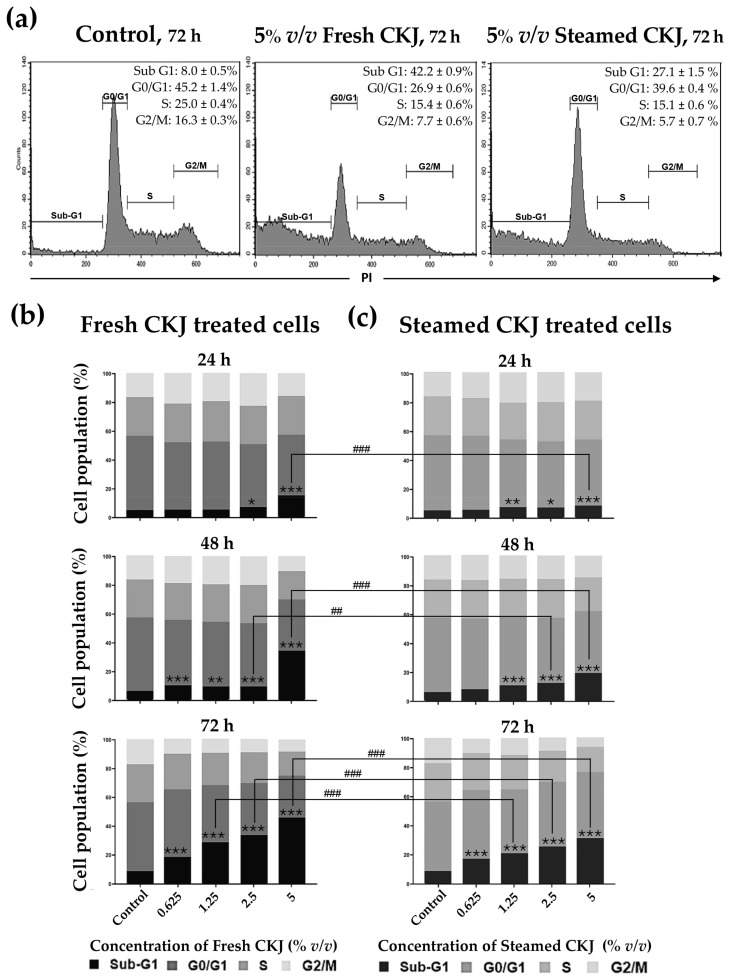
The distribution of U937 cells by cell cycle phase after treatment with fresh or steamed CKJ for 72 h. The flow cytometry profiles are shown in histograms (**a**) and the percentage of cells that were treated with various concentrations of fresh CKJ (**b**) and steamed CKJ (**c**) in the Sub G1, G0/G1, S, and G2/M phases are presented. The significance level of the tested sets was compared with a control (normal saline) set (* *p* = 0.05, ** *p* = 0.01, and *** *p* < 0.001) and fresh CKJ-treated cells were compared with steamed CKJ-treated cells (## *p* = 0.01 and ### *p* < 0.001). Data are representative of at least three independent experiments.

In this study, the cell cycle progression was analyzed to investigate whether treatment with fresh and steamed CKJ led to the induction of U937 cell cycle arrest. As shown in Figure 2 and Figure S3, we found that CKJ-treated cells showed a significant increase in cell population in the sub-G1 area compared with the control, and the number of cells in this area increased with extended exposure time and increased CKJ concentration. The results showed that fresh CKJ induces more cell cycle arrest at sub-G1 than steamed CKJ. The difference is more pronounced at higher concentrations of CKJ.

### 3.4. Effects of CKJ on U937 Cell Apoptosis

**Figure 3:**
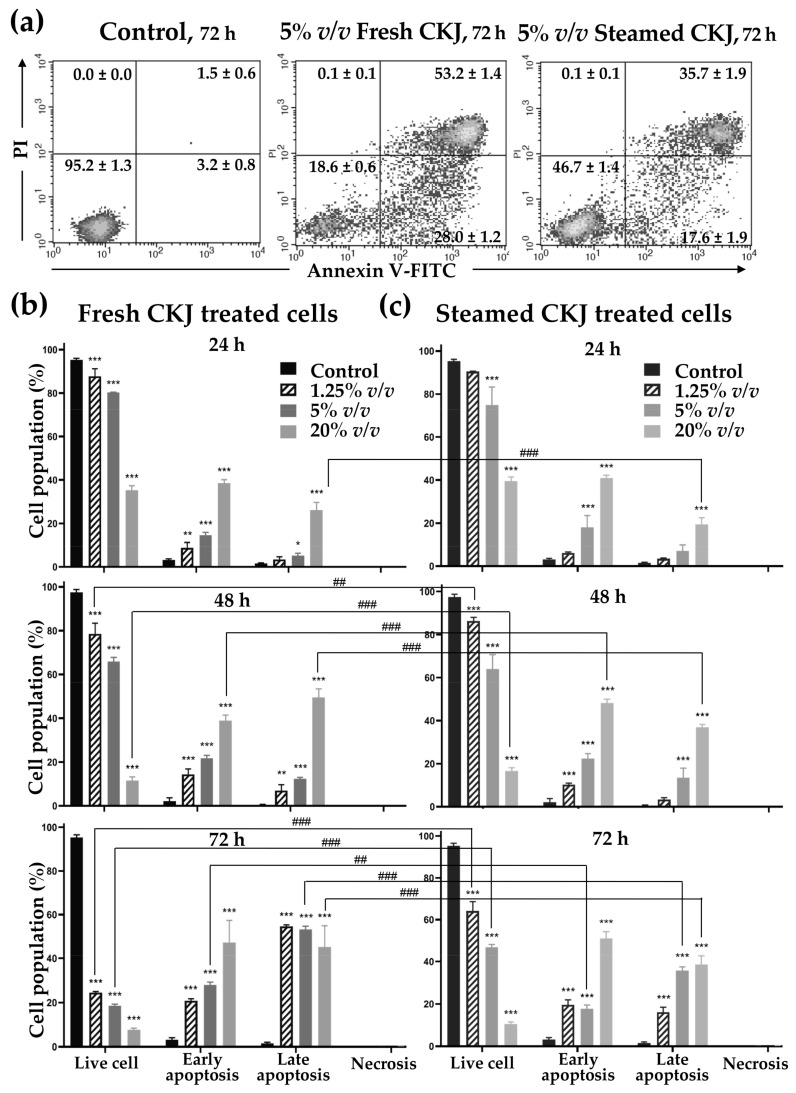
Induction of U937 cell apoptosis by fresh and steamed CKJ. The positive cells were quantified using a flow cytometer (lower left: live, lower right: early apoptosis, upper right: late apoptosis, upper left: necrosis) (**a**) The percentage of cells in each group was calculated (**b**,**c**). The significance level of the tested sets was compared with a control (normal saline) set (* *p* = 0.05, ** *p* = 0.01, and *** *p* < 0.001) and fresh CKJ-treated cells were compared with steamed CKJ-treated cells (## *p* = 0.01 and ### *p* < 0.001). Data are representative of at least three independent experiments.

To study the mechanism of CKJ that leads to the induction of U937 cell death, an apoptosis assay was performed. CKJ-treated U937 cells were examined using flow cytometry. The results are shown in Figure 3 and Figure S4. It is clearly seen that the percentage of apoptotic cells increased significantly with increased concentration of CKJ, compared with the control. The ratio of apoptotic cells increased gradually to more than 80% after 72 h of treatment with 20% *v*/*v* fresh and steamed CKJ. At higher concentrations of CKJ, more cells were in the late apoptotic stage. It is worth noting that no cell appeared to have died by necrosis even at a concentration of CKJ as high as 20% *v*/*v*.

### 3.5. Effect of CKJ on Apoptotic Protein Expression

**Figure 4:**
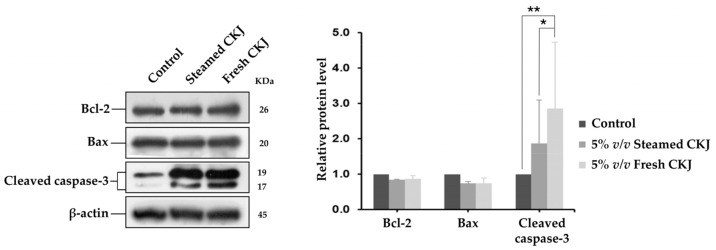
Western blot analysis for cleaved caspase-3, Bcl-2, and Bax using lysates from U937 cells exposed to fresh and steamed CKJ for 72 h (**left**). The graphs show the densitometric analysis, normalized against β-actin (**right**). (* *p* = 0.05 and ** *p* = 0.01). Data are representative of at least three independent experiments.

To examine the role of the apoptotic signaling cascade in U937 cells that were triggered by CKJ, the activation of pro-apoptotic and anti-apoptotic proteins, including cleaved caspase-3, Bcl-2, and Bax were analyzed. In this study, we found that treatment with both fresh and steamed CKJ triggers apoptosis in U937 cells and increases the amount of cleaved caspase-3, whereas the amounts of Bcl-2 and Bax were not altered (Figure 4 and Figure S5).

## 4. Discussion

In this study, we reported total glucosinolate and isothiocyanate contents of juice prepared from fresh and steamed Chinese kale leaves. The glucosinolate content in Chinese kale leaves shown in this study is about 3-fold higher than that reported by Hennig et al. [20]. The difference in the number of glucosinolates obtained in these studies is probably a result of different extraction and determination methods. Also, the variation in profiles and levels of both glucosinolates and isothiocyanates in Brassica plants is influenced by plant cultivars, growth and harvest conditions, storage conditions, etc. [21]. Although Chinese kale can be eaten raw, it is more popular when cooked by stir fry or blanching. Regarding the effect of cooking, the changes of glucosinolate and isothiocyanate contents in Brassica vegetables after processing and cooking can be unpredictable because they are influenced by many factors, such as the nature of vegetables, processing or cooking methods, cooking temperature, and duration. These conditions result in different degrees of leaching effect, degradation of the metabolites, and inactivation of myrosinase enzymes [22]. In this study, we chose steaming as a cooking method to prevent the leaching of active ingredients into the water. Since it has been reported that steaming for >7 min almost completely inactivates myrosinase enzymes, [23] therefore, we expected to see a higher amount of glucosinolates in steamed CKJ compared to fresh CKJ because they could not be converted to isothiocyanates. For the same reason, isothiocyanate contents were supposed to be higher in fresh CKJ than in steamed CKJ. Previously, there have been several conflicting reports on the effect of cooking on glucosinolate contents in cruciferous vegetables. Studies in a range of cruciferous vegetables have demonstrated the change of glucosinolates to various degrees ranging from +12.4 to −70.3% after steaming for 2–15 min [24,25,26]. However, some studies reported no significant change in total glucosinolates after steaming [23,27,28]. In our case, the equivalent amount of glucosinolates in fresh and steamed CKJ can be explained by the hypothesis that the slow rate of glucosinolate hydrolysis compensates for the thermal degradation of glucosinolates. Moreover, the presence of isothiocyanates in the steamed CKJ indicated that myrosinase enzymes in Chinese kale are not completely inactivated. Baenas and co-workers have shown that isothiocyanates were still present in the broccolini and kale samples after steaming for 15 min [24]. The increase in isothiocyanates in lightly cooked samples has been previously reported [29,30]. These findings suggest that the heat stability of myrosinase enzymes in different cruciferous vegetables is not similar. Additionally, the increase in isothiocyanates in cooked samples may be partly attributed to increased extractability due to the soft matrix.

Recently, glucosinolate contents of Chinese kale have been identified. Edible parts of Chinese kale have been shown to contain thirteen glucosinolates, including glucoraphanin and gluconasturtiin, which are precursors to the important isothiocyanates, sulforaphane, and phenetyl isothiocyanate [13,31]. Sulforaphane and phenetyl isothiocyanate have been recognized as potential isothiocyanates with anticancer activity. They have the ability to inhibit cell growth regardless of the origin of cancer cells [32]. Herein, we demonstrate, for the first time, that Chinese kale possesses a potential anti-leukemic effect. The results showed that the anti-leukemic effect of fresh CKJ is more pronounced than steamed CKJ. However, based on our findings, glucosinolates in fresh and steamed CKJ are not significantly different and isothiocyanates in steamed CKJ are higher than in fresh CKJ. These suggested that the anti-leukemic activity of CKJ was not primarily a result of glucosinolates and isothiocyanates but rather the overall activity of phytochemicals found in Chinese kale. According to Wang et al., kale leaves are high in vitamin C and kaempferol-derived flavonols [31]. These two ingredients have been shown to be effective in decreasing the viability of leukemic cells by apoptosis induction [33,34]. Several studies have reported that cooking processes, such as boiling, steaming, and high-power microwaving diminish anti-oxidant capacity and lead to the loss of many components in cruciferous vegetables, including vitamin C, tocopherols, carotenoids, phenolic compounds, and isothiocyanates [35,36,37]. The higher anti-leukemic activity of fresh CKJ compared to steamed CKJ is likely because fresh CKJ can retain most of its bioactive compounds. Accordingly, it can be inferred that consuming fresh vegetables would be more advantageous than cooked vegetables regarding the anticancer effect.

Characteristic features of leukemic cells are a blockade of differentiation at a distinct stage in cellular maturation and a loss of a normal function of the cell death program; therefore, induction of cell differentiation and apoptosis are considered plausible approaches for the treatment of leukemia [38]. Several studies have suggested that the induction of cell differentiation in cancer cells is tightly coupled to cell cycle arrest at G1 and G2/M, which are the cell cycle checkpoints during cell division, whereas the sub-G1 peak reflects the cell debris from apoptotic and necrotic cells [39,40]. Therefore, based on our results, it can be concluded that CKJ could not induce U937 cell differentiation, but instead direct cells into an apoptotic pathway.

Recently, research studies have focused on the potential of glucosinolates and isothiocyanates as chemopreventive agents based on their ability to target multiple mechanisms within the cell and thus control carcinogenesis [41,42]. These studies revealed some mechanisms involving apoptosis induction, including the generation of reactive oxygen species (ROS), upregulation of caspase-9 and Bax and downregulation of Bcl-2, and B-cell lymphoma-extra large protein (Bcl-xl), which are proteins that control the intrinsic apoptosis pathway. In this study, we found that treatment with CKJ leads to increased activation of caspase-3, which is a common executioner of apoptosis without alteration of Bcl-2 and Bax. These indicated that apoptotic induction of CKJ in the U937 cell line is modulated by other proteins in the Bcl-2 family or maybe other pathways. Gao and co-workers have reported that in U937 cells treated with phenethyl isothiocyanate, myeloid leukemia 1 protein (Mcl-1) is downregulated, whereas Bcl-2 and Bax are unaffected, and cleavage/activation of caspase-3, -8, and -9 was significantly increased [43]. Isothiocyanates can also exert their anti-leukemic effects through the induction of oxidative stress and extrinsic apoptotic pathway as suggested by Wang and co-workers [44]. The limitation of this study is that only proteins in the Bcl-2 family associated with the intrinsic apoptotic pathway are studied. Other apoptosis regulator proteins should be investigated for a better understanding of the antileukemic roles of Chinese kale.

A recent study on a *Brassica* vegetable in the same species as Chinese kale has shown that, besides apoptosis induction, the vegetable extract can induce the production of an anticancer cytokine (tumor necrosis factor-α) in HeLa and HepG2 cells [45]. In addition, the described study revealed selective cytotoxicity toward cancer cells while causing less harm to normal cells. Recently, it has been shown that bitter taste receptors or type 2 taste receptors (T2Rs) are associated with the risk of developing cancer and cancer prognosis [46]. There has been evidence that T2Rs were expressed and functionally active in leukemic cells (OCIAML3 and THP-1) [47]. Targeting such receptors could be the way for the development of leukemia therapies. Polyphenols and glucosinolates are the primary anticancer substances present in *Brassica* plants that possess a binding affinity for T2Rs [48]. Therefore, further studies that explore the molecular mechanisms of the toxic effects of Chinese kale on leukemic cells will be valuable. Moreover, the application of Chinese kale to other types of cancer is expected to be useful in developing cancer prevention and therapeutic strategies.

## 5. Conclusions

We have revealed that the cooking process, especially steaming, barely alters levels of both glucosinolates and isothiocyanates in Chinese kale, noting that the effect on other bioactive compounds was not evaluated. This study is the first demonstration that CKJ significantly impairs the proliferation of leukemic cells (U937) and triggers cell death through the induction of apoptosis. Fresh CKJ was more effective than steamed CKJ in obliterating leukemic cells. These findings suggest that it may be worthwhile to study the effects of Chinese kale on leukemic models and patients. Despite the potential health benefits of consuming Chinese kale, cooking with heat can deteriorate active ingredients and, thus, impair their function. Alternatively, Chinese kale extract could be further developed as a promising therapeutic option for leukemia that induces few adverse effects on patients or as an adjunct to other chemotherapy drugs to help increase the efficacy of leukemia treatment.

## Figures and Tables

**Table 1:** Total glucosinolate and total isothiocyanate contents of Chinese kale juice.

Chinese Kale Juice Preparation	Total Glucosinolates (μmol Sinigrin/100 g FW)	Total Isothiocyanates (μg/100 g FW)
Fresh	157.30 ± 5.56 ^a^	118.25 ± 1.87 ^a^
Steamed	139.40 ± 6.06 ^a^	127.64 ± 1.79 ^b^

Note: FW is abbreviated for fresh weight. Values with the different superscripts within the same column represent significant differences at *p* < 0.05.

## Data Availability

Data are contained within the article.

## Supplementary Materials

The following supporting information can be downloaded at: https://www.mdpi.com/article/10.3390/foods12071471/s1, Figure S1: Standard curve of sinigrin. Figure S2: HPLC chromatogram of 1,3-benzodithiol-2-thione of both standard and Chinese kale juice samples. Figure S3: Representative flow cytometry histograms of cell cycle analysis of U937 cells after incubation with control (normal saline), fresh CKJ and steamed CKJ at different concentrations (0.625, 1.25, 2.5 and 5% *v*/*v*) for 24, 48 and 72 h. Figure S4: Representative flow cytometry histograms of apoptosis analysis of U937 cells after incubation with control (normal saline), fresh CKJ and steam CKJ at different concentrations (1.25, 5 and 20% *v*/*v*) for 24, 48 and 72 h. Figure S5: Uncropped image of Western blots for Figure 4. Lanes showed U937 cells were treated with normal saline (Cont), steamed CKJ (C), fresh CKJ (F) and blank (B) of three independent experiments. Table S1: Antibody lists.
