# Supplementary material for: Anti-Leukemic Effects on a U937 Cell Line of Fresh and Steamed Chinese Kale Juice and Their Pro-Apoptotic Effects via a Caspase-Dependent Pathway
Source: Foods. 2023 Mar 30;12(7):1471. doi: 10.3390/foods12071471 (PMC10094306; doi:10.3390/foods12071471)
Supplement: Supplementary file 1 [file foods-12-01471-s001.zip › foods-2222171-supplementary.pdf]

Supplementary Information

Anti-Leukemic Effects on a U937 Cell Line of Fresh and Steamed Chinese Kale Juice and Their Pro-Apoptotic Effects via a Caspase-Dependent Pathway

Siriphorn Pungpuag [1], Somchai Boonpangrak [2] and Yaneenart Suwanwong [3,*]

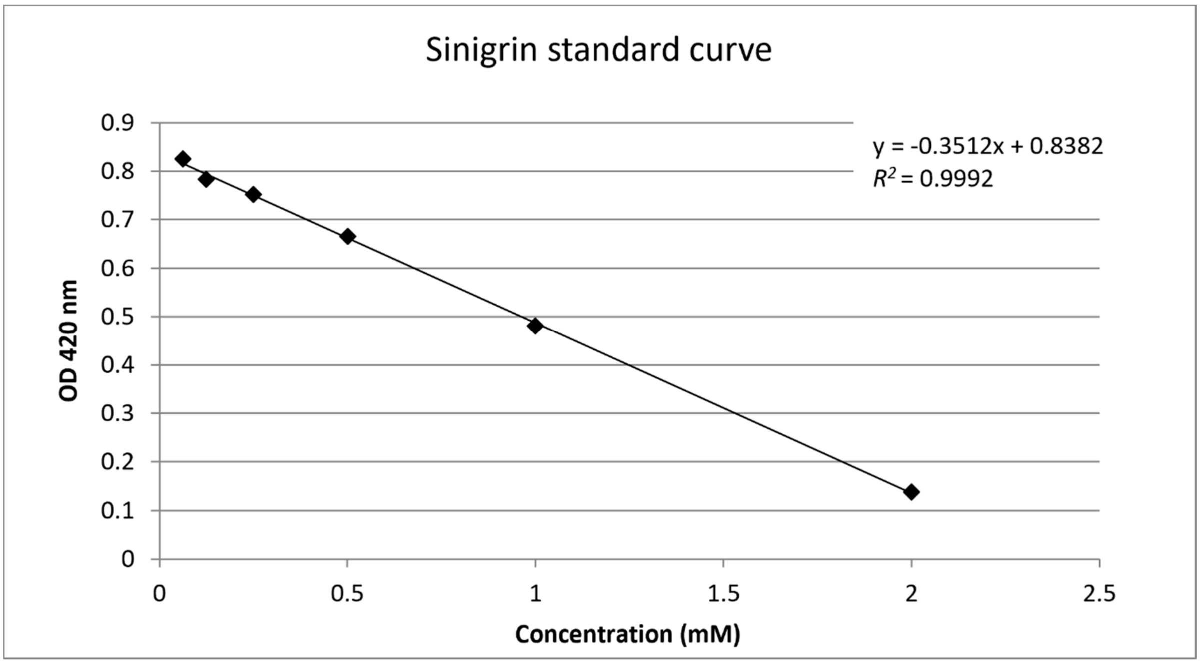

Supplementary Figure S1. Standard curve of sinigrin

A: Standard (1,3-benzodithiol-2-thione): 5 μg/mL

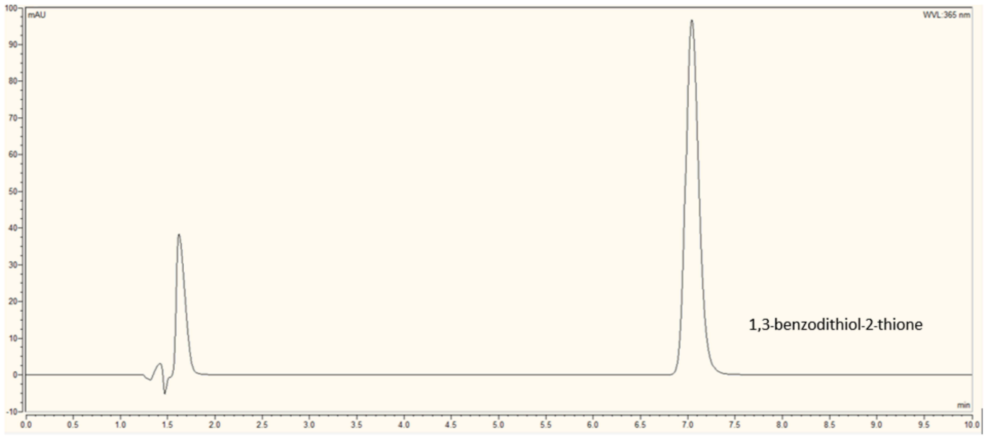

B: 1,3-benzodithiol-2-thione of fresh Chinese kale

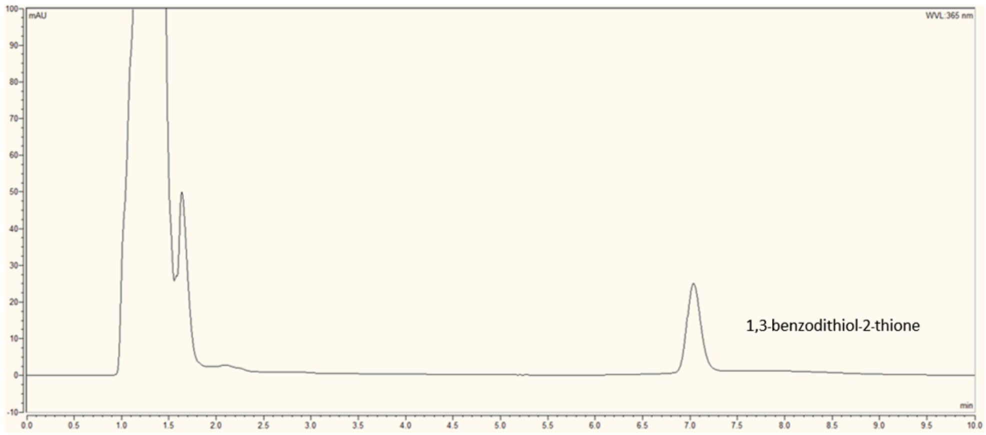

C: 1,3-benzodithiol-2-thione of steamed Chinese kale

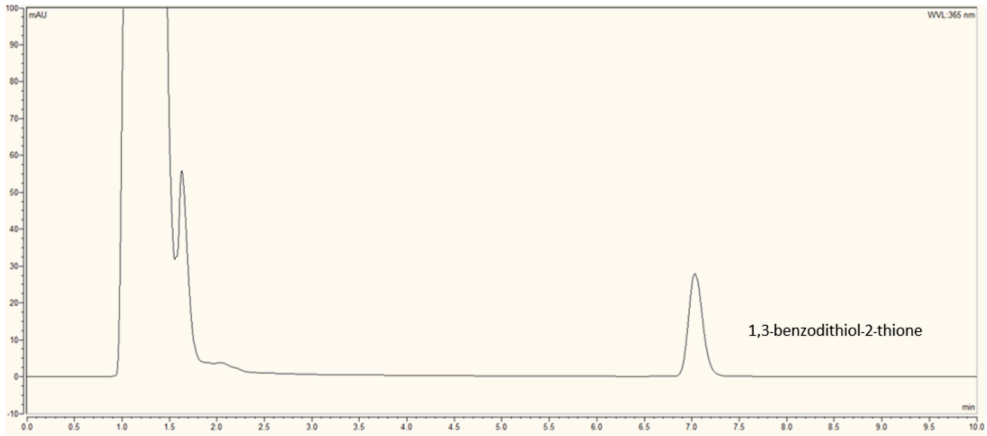

Supplementary Figure S2. HPLC chromatogram of 1,3-benzodithiol-2-thione of both standard and Chinese kale juice samples.

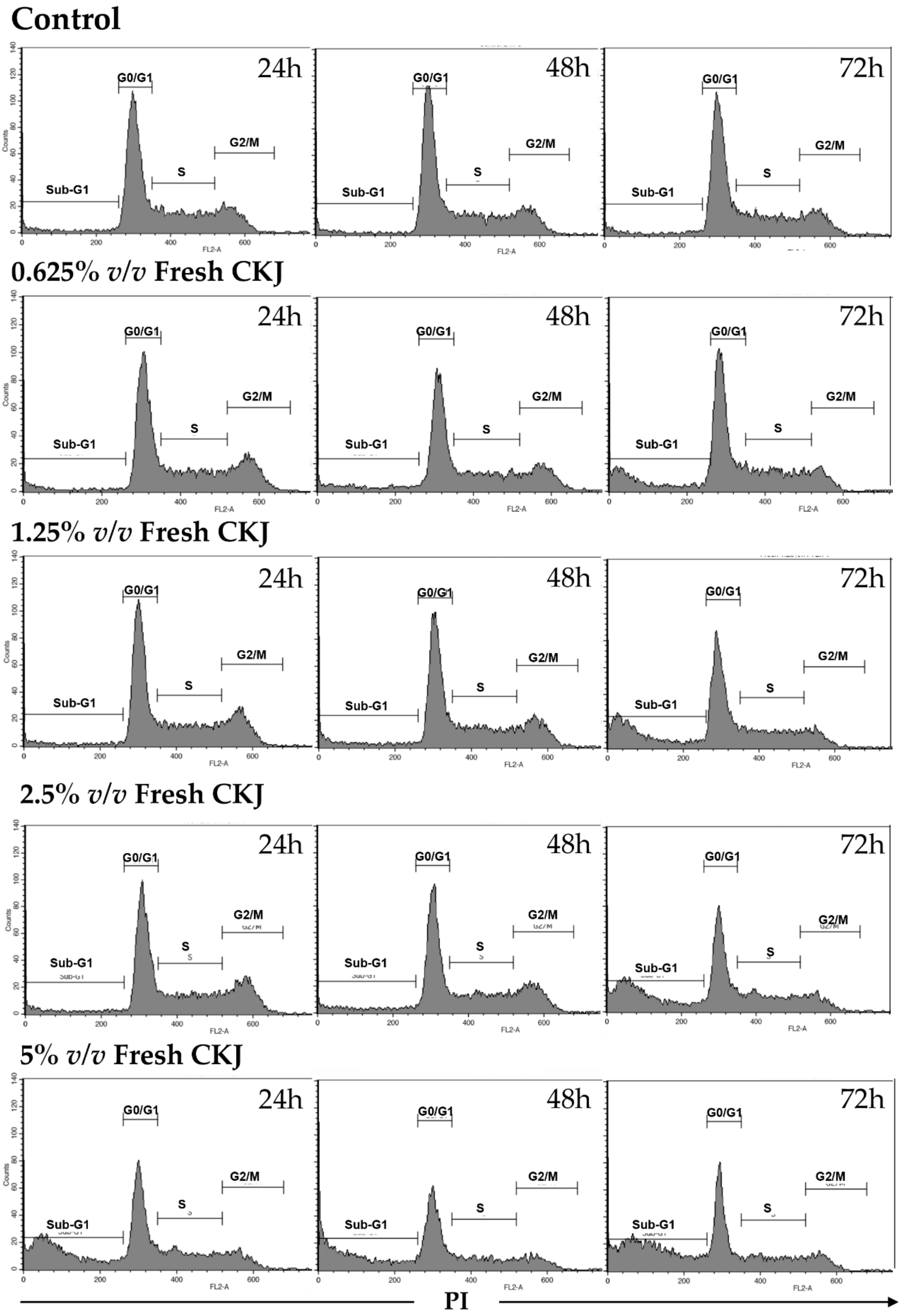

Control
0.625% v/v Fresh CKJ
1.25% v/v Fresh CKJ
2.5% v/v Fresh CKJ
5% v/v Fresh CKJ
24h
48h
72h
G0/G1
S
G2/M
Sub-G1
Counts
FL2-A
0
20
40
60
80
100
120
140
200
400
600
PI

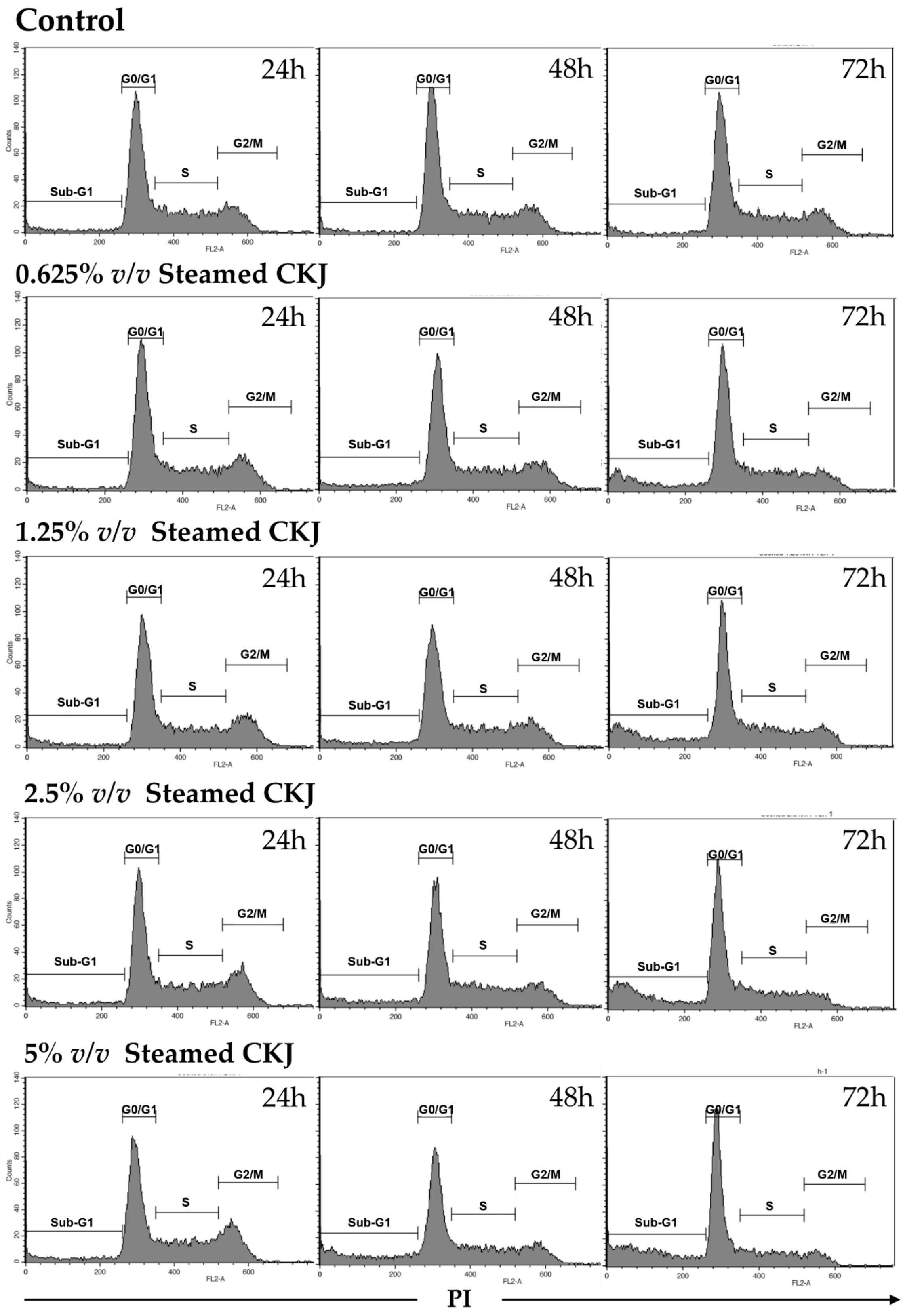

Supplementary Figure S3. Representative flow cytometry histograms of cell cycle analysis of U937 cells after incubation with control (normal saline), fresh CKJ and steamed CKJ at different concentrations (0.625, 1.25, 2.5 and 5% *v/v*) for 24, 48 and 72 h.

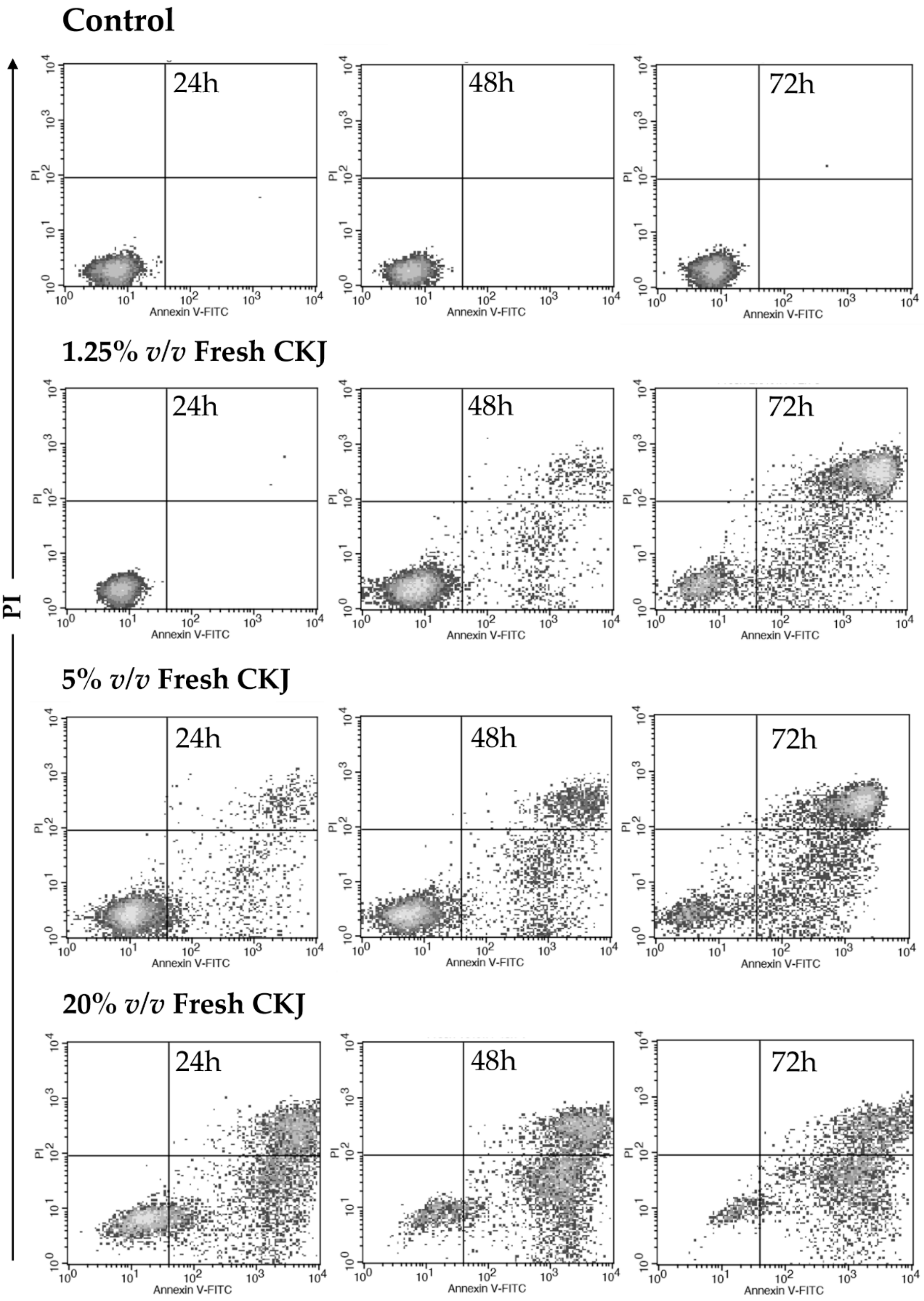

Control
1.25% v/v Fresh CKJ
5% v/v Fresh CKJ
20% v/v Fresh CKJ
24h
48h
72h
PI
Annexin V-FITC

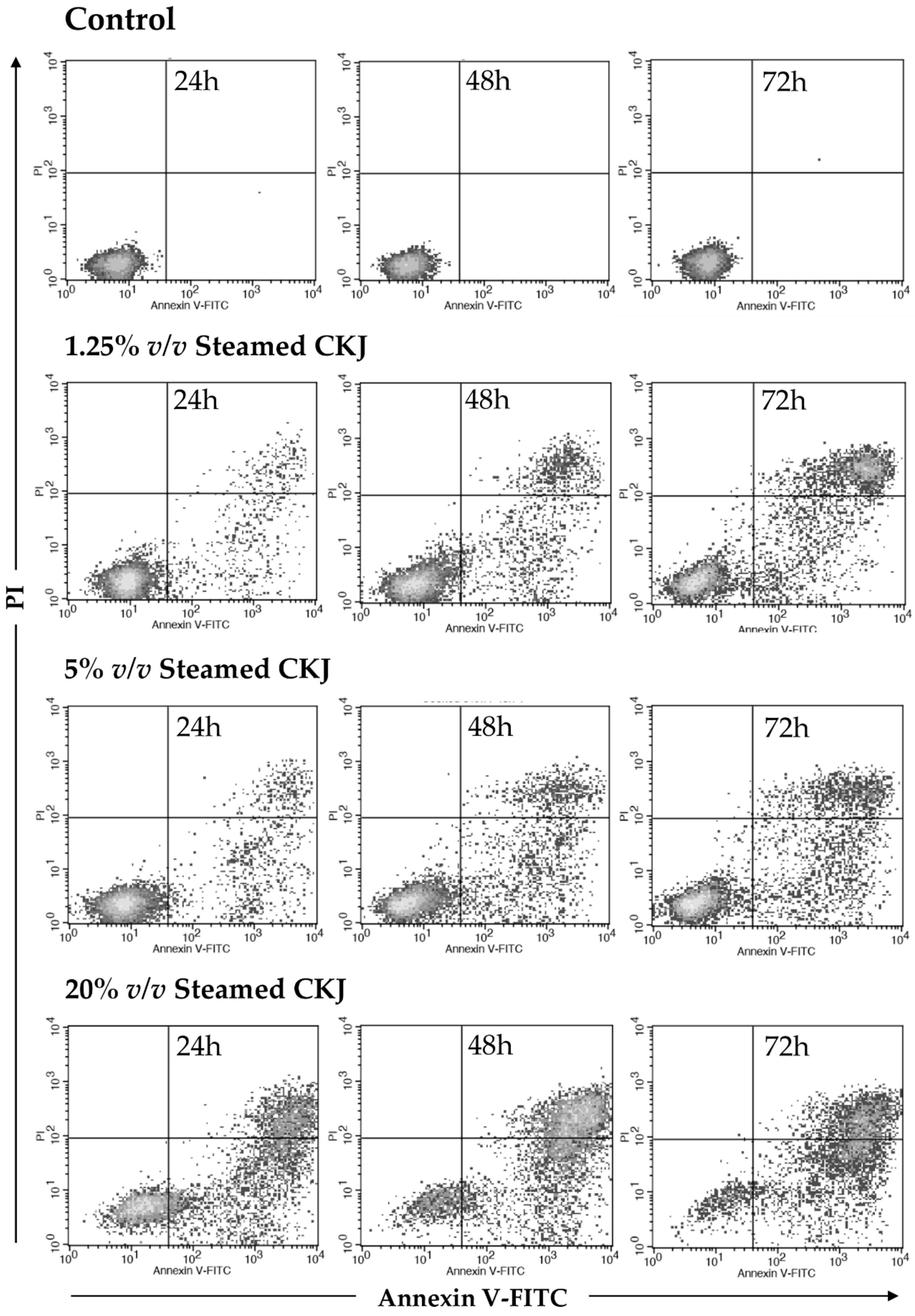

Supplementary Figure S4. Representative flow cytometry histograms of apoptosis analysis of U937 cells after incubation with control (normal saline), fresh CKJ and steamed CKJ at different concentrations (1.25, 5 and 20% *v/v*) for 24, 48 and 72 h.

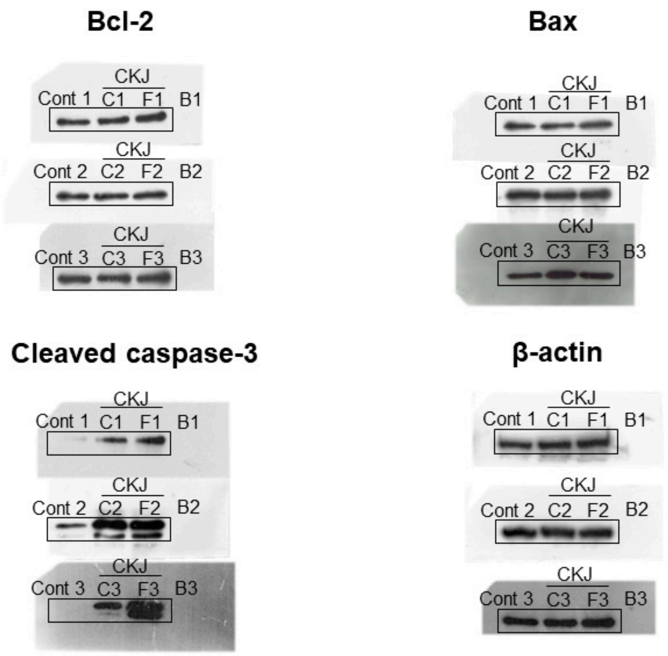

Supplementary Figure S5. Uncropped image of Western blots for Figure 4. Lanes showed U937 cells were treated with normal saline (Cont), steamed CKJ (C), fresh CKJ (F) and blank (B) of three independent experiments.

Supplementary Table S1. Antibody lists.

Antibody	Catalog No.	Source
Bcl-2	#4223	Cell Signaling Technology
Bax	#2772	Cell Signaling Technology
Cleaved caspase-3	#9664	Cell Signaling Technology
β-Actin	#4970	Cell Signaling Technology
Anti-rabbit IgG HRP	#7074	Cell Signaling Technology
